# Even modest prediction accuracy of genomic models can have large clinical utility

**DOI:** 10.3389/fgene.2014.00417

**Published:** 2014-11-28

**Authors:** Emily J. Dhurandhar, Ana I. Vazquez, George A. Argyropoulos, David B. Allison

**Affiliations:** ^1^Department of Health Behavior, Nutrition Obesity Research Center, Office of Energetics, University of Alabama at BirminghamBirmingham, AL, USA; ^2^Section on Statistical Genetics, Department of Biostatistics, University of Alabama at BirminghamBirmingham, AL, USA; ^3^Weis Center for Research, Institute of Obesity, Geisinger Health SystemDanville, PA, USA; ^4^School of Public Health, University of Alabama at BirminghamBirmingham, AL, USA

**Keywords:** genomics, prediction, clinical application, methods, statistical genetics

## Abstract

Whole Genome Prediction (WGP) jointly fits thousands of SNPs into a regression model to yield estimates for the contribution of markers to the overall variance of a particular trait, and for their associations with that trait. To date, WGP has offered only modest prediction accuracy, but in some cases even modest prediction accuracy may be useful. We provide an illustration of this using a theoretical simulation that used WGP to predict weight loss after bariatric surgery with moderate accuracy (*R*^2^ = 0.07) to assess the clinical utility of WGP despite these limitations. Prevention of Type 2 Diabetes (T2DM) post-surgery was considered the major outcome. Treating only patients above predefined threshold of predicted weight loss in our simulation, in the realistic context of finite resources for the surgery, significantly reduced lifetime risk of T2DM in the treatable population by selecting those most likely to succeed. Thus, our example illustrates how WGP may be clinically useful in some situations, and even with moderate accuracy, may provide a clear path for turning personalized medicine from theory to reality.

The hoped-for future of using genomics to implement personalized medicine that was to follow the completion of the Human Genome Project in 2003 has not yet fully come to fruition. Genome Wide Association Studies (GWAS) have identified variants associated with monogenic diseases that have led to substantial improvements in prediction and prevention, but methods for prediction of more complex polygenic diseases and traits have not been as forthcoming. However, thousands of variants associated with complex diseases have now been identified, high throughput sequencing technology is quickly advancing, and new statistical modeling techniques are being implemented to incorporate genetic information into prediction models. Even if the resulting predictive ability is only moderate to date, there are circumstances where certain methods may have substantial clinical utility in the near future.

Prediction of treatment response when treatment success is highly variable may be one such circumstance. Imagine a patient considering gastric bypass surgery for treatment of morbid obesity. On average, roughly 15% of patients lose all of their excess body weight with surgery. In addition, roughly 13% experience serious adverse events, and surgery is expensive. Alternatively, some patients experience far greater weight loss and experience no serious adverse events. In this scenario, the ability to test for an underlying genetic receptiveness to surgery-induced weight loss and side effects would be valuable.

Whole Genome Prediction (WGP) is an advanced statistical method that incorporates many thousands of SNPs into a regression model and, fitting them jointly, yields estimates for the contribution of all markers to the overall variance in a particular phenotype (Meuwissen et al., 2001). This approach may be particularly effective for modeling genetic risks for complex traits. For example, using single nucleotide polymorphisms identified as statistically significant using single marker regressions, those associated with height account only for 5% of the total variance, however, WGP accounts up to 83% of the total variance in height (Makowsky et al., 2011). Although WGP models have demonstrated a moderate degree of predictive accuracy when predicting the phenotype of humans who were not used to develop the predictive model (*R*^2^ of 0.15–0.36 for height, Makowsky et al., 2011), even this modest prediction accuracy may be of clinical value in some circumstances.

For illustrative purposes, we conducted a simulation in which WGP with modest prediction accuracy (predictive *R*^2^ = 0.07) was used to predict weight loss after bariatric surgery. Although this predictive *R*^2^ is low for typical predictive modeling standards, it is within a moderate range of what is typically achieved using WGP (Makowsky et al., 2011). The main outcomes of interest were prevention of Type 2 Diabetes Mellitus (T2DM) via surgery-induced weight loss, and risk of adverse events. A predictive decision tool would be especially useful for this scenario, as bariatric surgery is a high cost, invasive treatment with varied success and non-trivial risks, and limited family history information.

The simulated dataset was used to examine the utility of WGP in two different contexts. The impact of the prediction tool on the probability of adverse events and the probability of developing T2DM was determined in a realistic scenario where resources are limited in comparison to the number of eligible patients. For example, suppose there are a limited number of surgeons, operating rooms, and monetary resources that make it impossible to treat every eligible patient. We calculated outcomes with a large but finite amount of resources, in the context of a future scenario where genomic information is available for all patients. Total adverse event cases and cases of T2DM were determined at several τ, or “decision thresholds” of predicted weight loss, above which the patient is allocated to surgery, below which the patient is not allocated to surgery. We compared both outcomes at various τ to the current situation where no genomic test is used.

To simulate a bariatric surgery eligible population, a distribution of body mass index (BMI) was simulated from nationally representative height and weight data for adult males and females (Romero-Corral et al., 2008). We then assumed the portion of the distribution above a BMI of 35 was representative of the surgery eligible population. Using a typical bariatric surgery weight loss distribution, we estimated the final BMI for this population after surgery. A dose response relationship was used to describe the probability of future T2DM as a function of body weight (Narayan et al., 2007). The probability of an adverse event was also simulated, with baseline body weight and gender modifying the risk of an adverse event (Ortega et al., 2012). Finally, predicted weight loss was simulated with very moderate prediction accuracy (*R*^2^ = 0.07 for predicted vs. actual weight loss), to reflect realistic prediction accuracy.

We based our simulated data in distributions and parameters found in the literature. We initially simulated body measures for 150,000 humans, and selected only the candidates for gastric bypass (body mass index, BMI, equal or larger than 35). The candidates of gastric bypass were then assumed to have genetic testing for response to treatment.

We simulated 150,000 heights and weights drawing samples from a Multivariate Normal Distribution for height and logarithm of the weight (later transformed to weights). Outliers for weight (>180 kg or <38 kg) and height (<145 cm) were removed. Location and shape parameters for height were 160.6 ± 6.2 cm (mean ± standard deviation), 4.2 ± 0.19 log(kg) for log transformed weight, and 0.59 (co)variance, between height and log transformed weight. These parameters correspond to the mean and standard deviation for female descriptive statistics in the US population. Next, half of the data were randomly declared male, using a uniform distribution between 0 and 1, and a cutoff at 0.5 above which data were declared male. Male height and weight was modified by adding a constant to increase the mean. Males had an effect of increased height and weight of 13 cm and 9.4 kg, respectively, (thus, location parameter for final height and weight would be larger than originally determined). The parameters for the distribution of height and log transformed weight were found to match height and weight location and shape according to Romero-Corral et al. (2008).

Percentage of excess weight loss (PEWL) was also simulated following a normal distribution with parameters 63.0 ± 23.0. PEWL was forced to have an association of 0.59 with body weight. The centered and standardized weight variable was multiplied by 0.59, and added to a random residual multiplied by 0.85. Location and shape parameters of the resulting random variable were accommodated by adding and multiplying constants to achieve *N*(*PEWL*|63.0,23.0). The genetic prediction of the PEWL followed a normal distribution with parameters 63.0 ± 7.6 and has a correlation of 0.316 (i.e., *R*^2^ = 0.1), with actual PEWL. Recent results of genomic predictions show an *R*^2^ between unrelated individuals of 0.11 for height and a correlation of 0.27 (*R*^2^ = 0.073) for BMI with a family data set (Vazquez et al., unpublished data), which suggest that our correlation between predicted and actual values could be a reasonable assumption.

We performed calculations for 3592 subjects of the abovementioned population, to include only the people with body mass index of 35 or larger. A BMI of 35 was the cutoff considered for gastric bypass candidates. We considered an ideal BMI to be 25, and used this to enumerate ideal and excess body weight of each subject, using guidelines at the National Heart, Lung and Blood Institute (http://www.nhlbi.nih.gov/guidelines/). With the excess body weight and PEWL data, we derived weight after surgery and BMI (by subtracting excess weight^*^PEWL from weight).

Next, we generated a function to map BMI to probabilities of diabetes for each BMI value. This mapping was intended to follow values found in the literature (Ortega et al., 2012). Based on a range of BMI from 20 to 55, we generated a function multiplying a centered and standardized BMI by 0.8. The probability of diabetes corresponding to BMI values was the empirical cumulative distribution function (CDF) of this function (Narayan et al., 2007, Table 1, adult age range). This map follows the probabilities estimated in Narayan et al. and makes the transition between BMI categories smooth. The probability of diabetes before and after the surgery was derived with the same function. Finally, we accounted for probability of adverse events as a function of both, BMI and gender, using the function described in Ortega et al. (2012). This function was used to calculate the probability of adverse events for each subject if they were to have treatment. The resulting dataset was used to calculate our outcomes of interest.

## Computation of outcomes

1. For a given threshold of predicted weight loss the probability of diabetes and adverse events was calculated for the entire population. To calculate the population level (both treated and untreated) probability of diabetes and adverse events at each threshold.
  $$P(C)|\tau =\frac{\left(\Sigma_{i:\alpha_{i}>\tau }\frac{P(C_{it})}{n_{t}}\right)+\left(\Sigma_{i:\alpha_{i}<\tau }\frac{P(C_{iu})}{n_{u}}\right)}{2},$$
  where *P*(*C*) is the probability of a case in the population, *P*(*C_it_*) is the probability of a case for a given individual *i* if treated, *n_t_* is the number of individuals in the treated population, *P*(*C_iu_*) is the probability of a case for a given individual *i* if not treated, *n_u_* is the number of individuals in the untreated population, *α_i_* is the predicted weight loss for a given individual *i*, and τ is the decision threshold of predicted weight loss.
2. In the context of finite resources we computed the number of additional cases prevented, *N*, by using the genomic test compared to not using the test. For this we ordered the data from higher to lower predicted PEWL, and selected individuals for surgery only the subjects above the given threshold.
  $$N|\tau =1000(P(C_{iu})-1000\left(\Sigma_{i:\alpha_{i}>\tau }\frac{P(C_{it})}{nt}\right)$$
  where 1000 represents the number of surgeries that can be performed with $20 million in resources, *P*(*C_iu_*) is the probability of a case in the untreated population, *P*(*C_it_*) is the probability of a case for a given individual in the treated population, *n_t_*is the number of individuals in the treated population, *α_*i*_* is the predicted weight loss for a given individual *i*, and τ is the decision threshold of predicted weight loss.
3. We did the previous calculation (1 and 2) for a sequence of thresholds from 55 to 80 of predicted weight loss.
4. For every subject we calculated the probability of diabetes with and without surgery conditional in the genetic predictor of the PEWL with spline functions.

**Figure 1:**
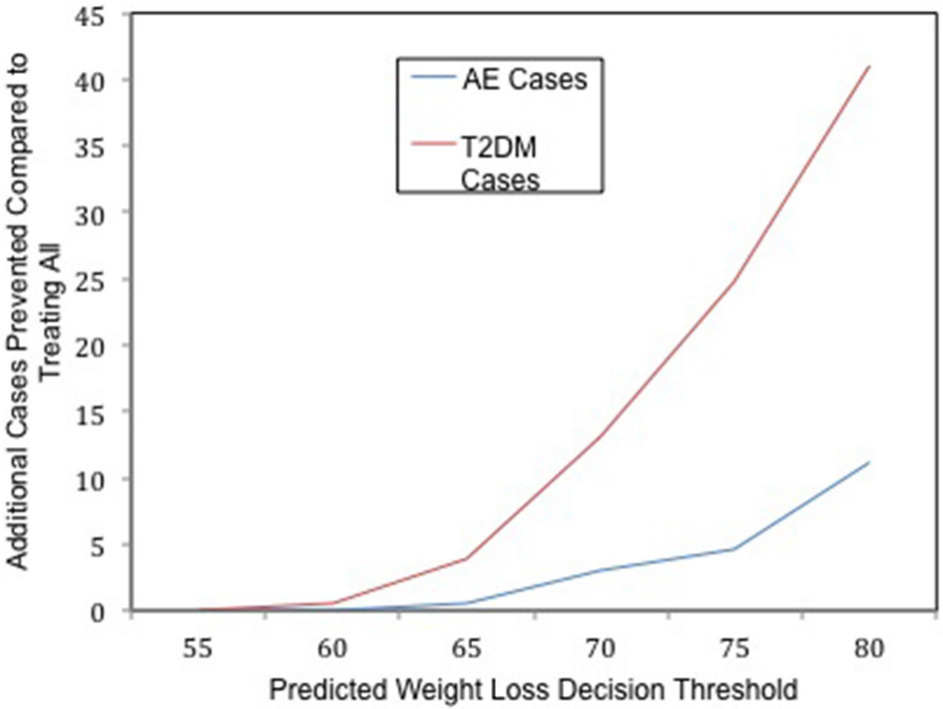
**The clinical utility of whole genome prediction**. The number of additional T2DM (red) and Adverse Event (AE, blue) cases prevented as a result of implementing the genomic test at a given decision threshold of predicted weight loss in the context of limited resources.

The difference in outcomes with and without the use of genomic testing is striking even though, in our hypothetical model, the genomic predictions only explain about 7% of the variance in the actual outcomes. When the genomic test is not implemented, and all patients receive surgery on a first come, first serve basis regardless of their likelihood of successful treatment response, the probability of an adverse event is 0.049 in our simulated population. If the genomic test is implemented, the predicted probability of an adverse event drops in the treated population as τ increases (see Figure 1). The adverse event rate drops when the test is used because the risk of an adverse event is positively associated with pre-surgery body weight, and the test selects those who are most likely to lose a greater percent excess weight, who also tend to be patients who ironically weigh less pre-surgery (Ortega et al., 2012).

Similarly, the lifetime risk of T2DM is 0.28 in the surgery eligible population if the test is not implemented, whereas if the test is implemented, the lifetime risk of T2DM in the treated population decreases as τ increases. By using the genomic test to carefully select participants to receive one of the limited number of surgeries, and selecting those most likely to lose most of their excess body weight, the population that receives the greatest preventative benefit is allocated to receive surgery. Treating only those patients who are predicted to lose at least 80% of their excess body weight will prevent an additional 41 future cases of T2DM (see Figure 1), compared to not using the tool and treating everyone in a first come, first serve manner with an equal amount of finite resources. Thus, using the genomic tool reduces the overall risk of T2DM in the population eligible to receive surgery in this context.

WGP may also be used by individuals and healthcare providers for making treatment decisions. Recall our patient considering bariatric surgery. Imagine her probability of T2DM if she does not receive surgery is 0.62, and her predicted weight loss is 84% of excess body weight. Having failed to lose weight through conventional methods, she decides that since her predicted weight loss is relatively high, the surgery may be worth the cost and risk. Suppose, in a different scenario, her probability of T2DM is still relatively high at 0.58, but with a predicted excess weight loss of only 57%, she is less confident the surgery will be worth the cost and risk given she is relatively healthy at present.

WGP thus has potential to be useful for allocating treatment and to aid in individual decision-making, even with modest prediction accuracy. The implementation of this information brings forth many ethical complexities, that would demand careful consideration; much like the impact of any genetic test. Even so, WGP is an example of an advanced statistical method that may be clinically useful for using genetic information for prediction of treatment response and complex disease traits, and is an example of progress following the completion of the Human Genome Project that brings us closer to turning personalized medicine from theory to reality.

### Conflict of interest statement

The authors declare that the research was conducted in the absence of any commercial or financial relationships that could be construed as a potential conflict of interest.
